# Supplementary material for: Water quality improvements offset the climatic debt for stream macroinvertebrates over twenty years
Source: Nat Commun. 2019 Apr 26;10:1956. doi: 10.1038/s41467-019-09736-3 (PMC6486586; doi:10.1038/s41467-019-09736-3)
Supplement: Supplementary file 1 — Supplementary Information [file 41467_2019_9736_MOESM1_ESM.pdf]

Supplementary Information

Water quality improvements offset the climatic debt for stream macroinvertebrates over twenty years

Vaughan & Gotelli

Supplementary Methods

Loglinear analysis for selecting the Markov model structure

Following Caswell[1], we fitted a hierarchy of models in which transition probabilities are homogenous across space and time, vary only in space or time, or are allowed to vary across both space and time. A comparison of these different models based on likelihood ratio tests or the Akaike Information Criterion (AIC) indicates whether there is evidence of heterogenous transition probabilities in space, time or both, which may need to be included in the final Markov chain model if the effect appears to be biologically relevant[2].

In English and Welsh rivers, the major sources of spatial variation in macroinvertebrate community structure are: i) the longitudinal gradient from upland, fast-flowing headwaters to lowland rivers, and ii) the contrast between rural and urban locations[3]. We therefore ran the loglinear analysis twice, once comparing upland and lowland rivers, and once for rural and urban. In both cases, the 3067 sampling locations were divided into two near-equal sized groups (n = 1533 and 1534), maximising the contrast between the river types, whilst maintaining the sample size to estimate transition probabilities. Loglinear models failed to converge in some instances for five biological classes; hence the analysis was restricted to the 3-class versions. The analysis was run for the classifications generated by the four clustering methods (two distance coefficients x two clustering algorithms).

We distinguished upland and lowland rivers by performing a principal component analysis using altitude, slope and distance from source of each sampling location. We extracted the scores from the first principal component (which explained 57% of the total variance) as a measure of how ‘upland’ a location was, and used the median score to split the locations into two groups. The urban–rural split was based on the median coverage of the WFD catchment by urban or suburban land cover, calculated from the 25m resolution UK Land Cover Map 2000 (ref. 4).

Supplementary figures

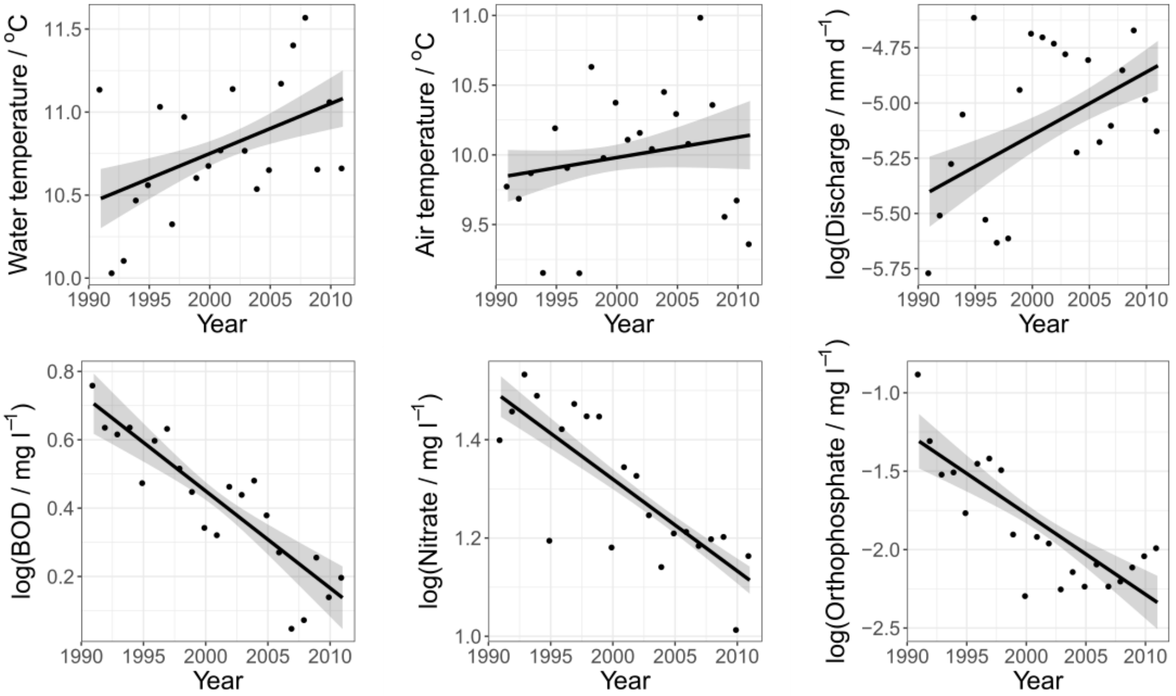

Supplementary Figure 1. Observed changes in mean environmental conditions across the 3067 sampling locations over the study period (n = 26,103). Regression lines fitted using generalised least squares (± standard error). Source data are provided as a Source Data file.

a

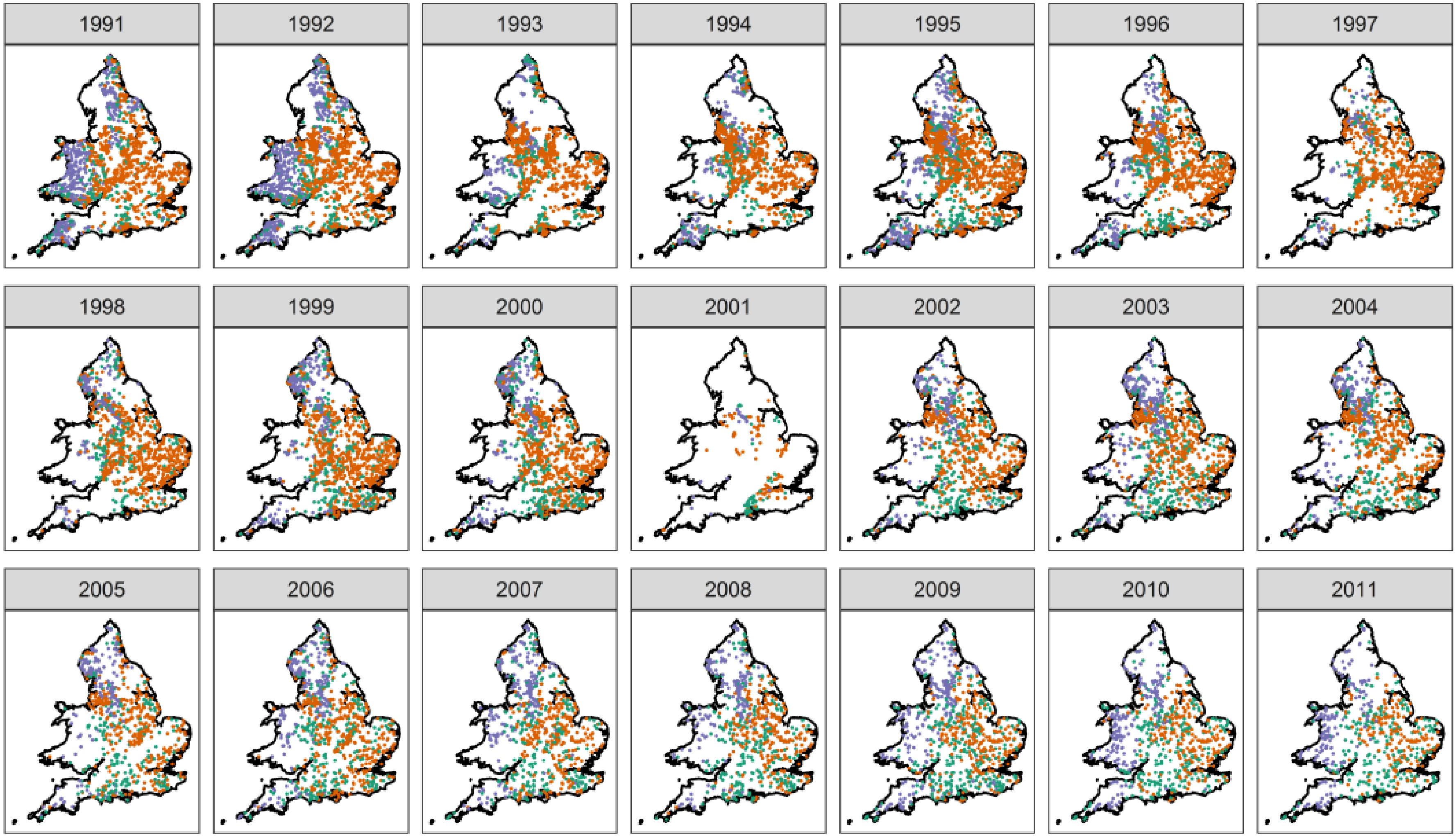

1991
1992
1993
1994
1995
1996
1997
1998
1999
2000
2001
2002
2003
2004
2005
2006
2007
2008
2009
2010
2011

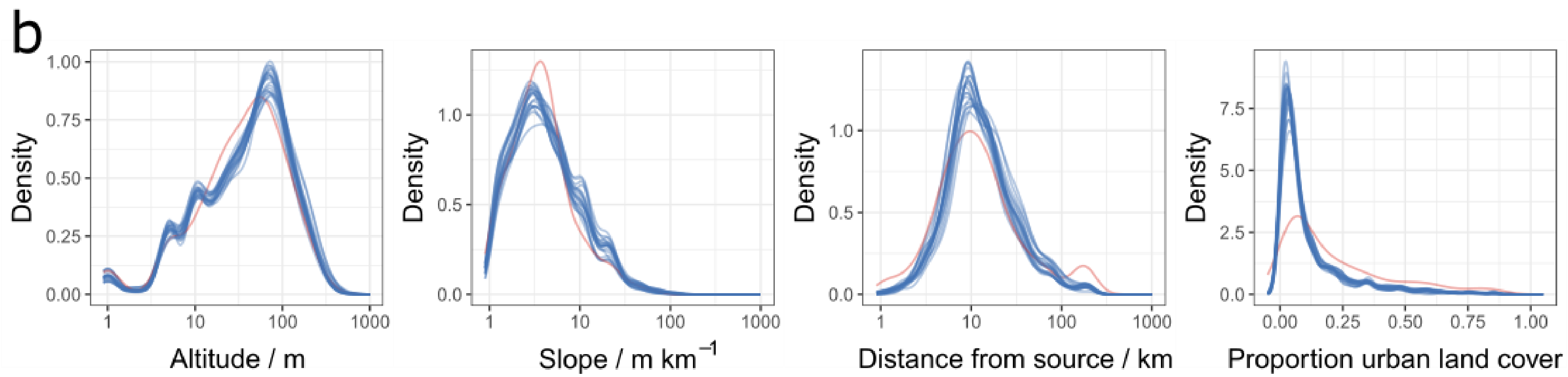

Supplementary Figure 2. The distribution of locations sampled in each year of the study: (**A**) across England and Wales, classified into the three biological classes, and (**B**) topographically, based on their elevation above sea level, slope of the channel, distance downstream from the source and proportion of their catchments under urban land cover. The colour scheme in Part **A** follows Fig. 1 in the main paper. Part **B** uses density plots, calculated for each of the 21 years and then superimposed. Sampling was curtailed in 2001 (red lines in part **B**) as part of measures to minimise risk of spreading foot-and-mouth disease amongst livestock, but data were retained in the analysis. Maps contain OS data © Crown copyright and database right (2017).

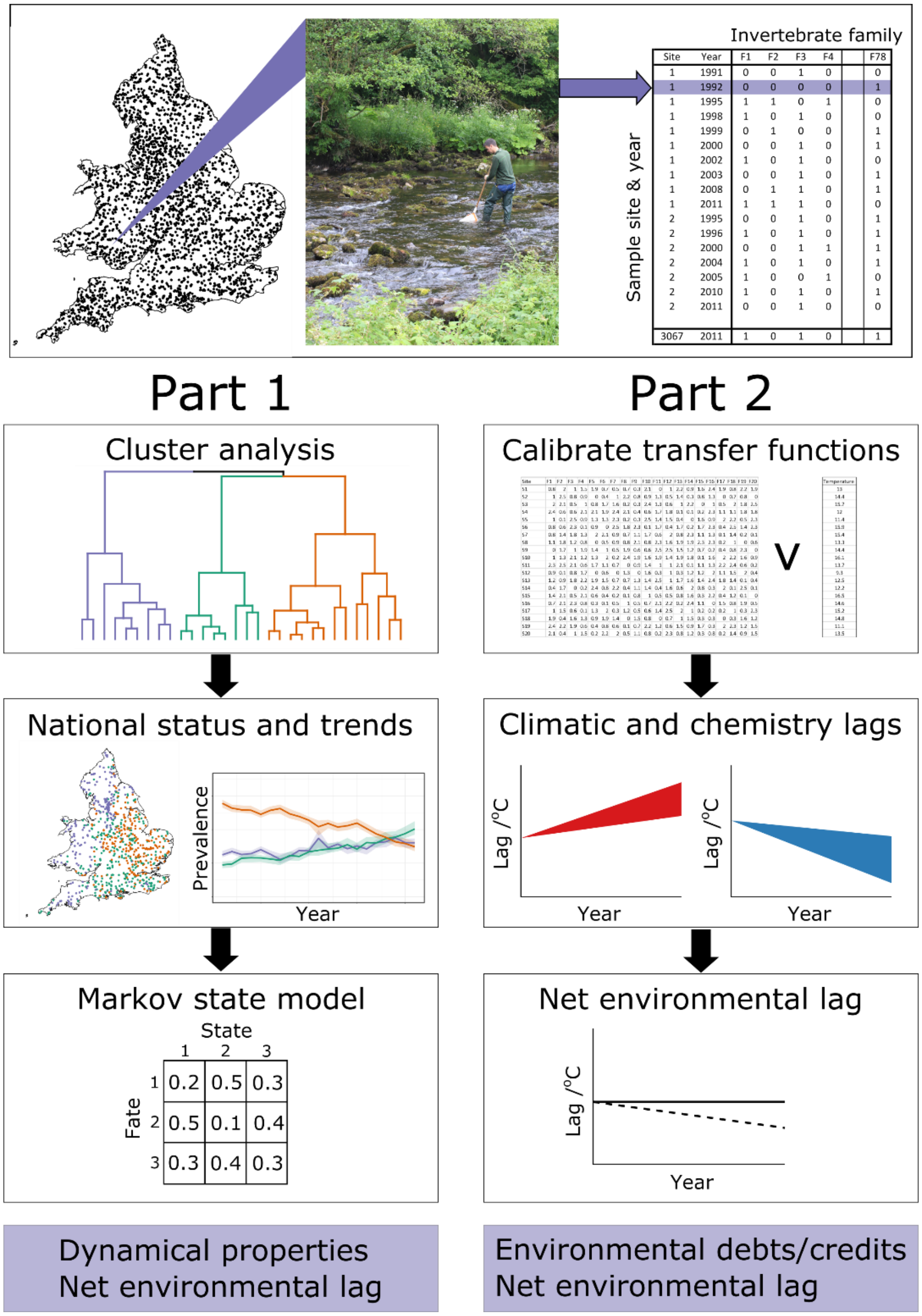

Supplementary Figure 3. Overall workflow for the study. Top row: macroinvertebrate data were collected from 3067 locations using a standard kick sampling protocol, before being identified to family-level. Part 1 of the study involved classifying the macroinvertebrate communities and assessing national-scale trends, before using a Markov state transition model to evaluate the dynamical properties of the system and overall environmental lag. Part 2 involved calibrating transfer functions to estimate environmental variables (e.g. water temperature) from the invertebrate community, before estimating environmental lags (credit or debt) for individual variables, and the net environmental lag between pairs of variable (e.g. water temperature and BOD). Maps contain OS data © Crown copyright and database right (2017).

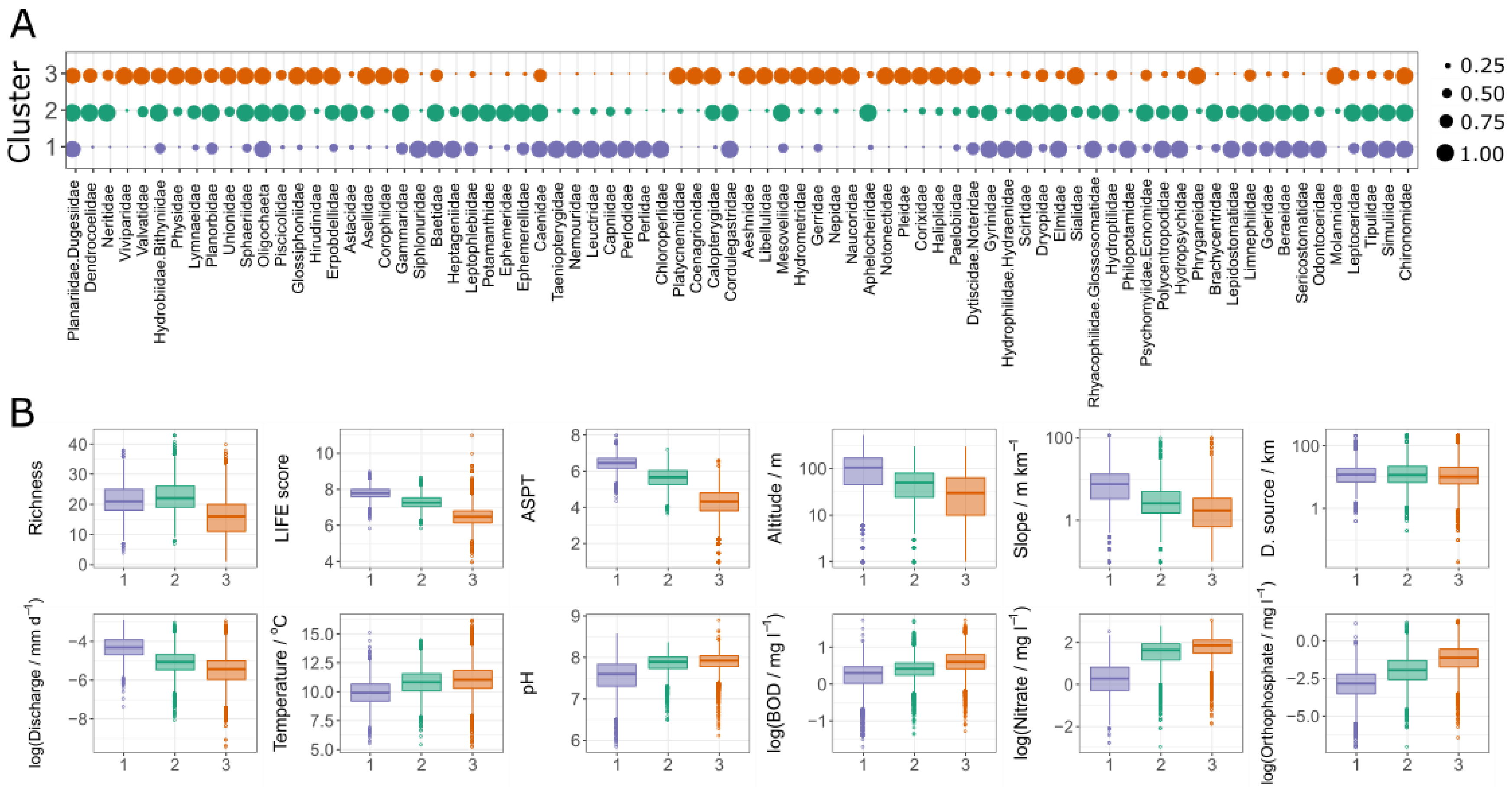

Supplementary Figure 4a. Structure of the three biological classes used in the main text (Ward's clustering, Jaccard dissimilarities) and their relationship with community-level metrics and environmental variables. **(A)**. The relative prevalence of the 78 macroinvertebrate taxa across the three clusters (maximum prevalence for each taxon = 1); taxonomy follows Vaughan and Ormerod[5]; **(B)**. The number of taxa, preference for faster-flowing conditions (LIFE score[6]) and average sensitivity to organic pollution (ASPT[7]), topography, hydrology, temperature and water chemistry for macroinvertebrate samples in the three classes. The colour scheme follows Fig. 1 in the main paper.

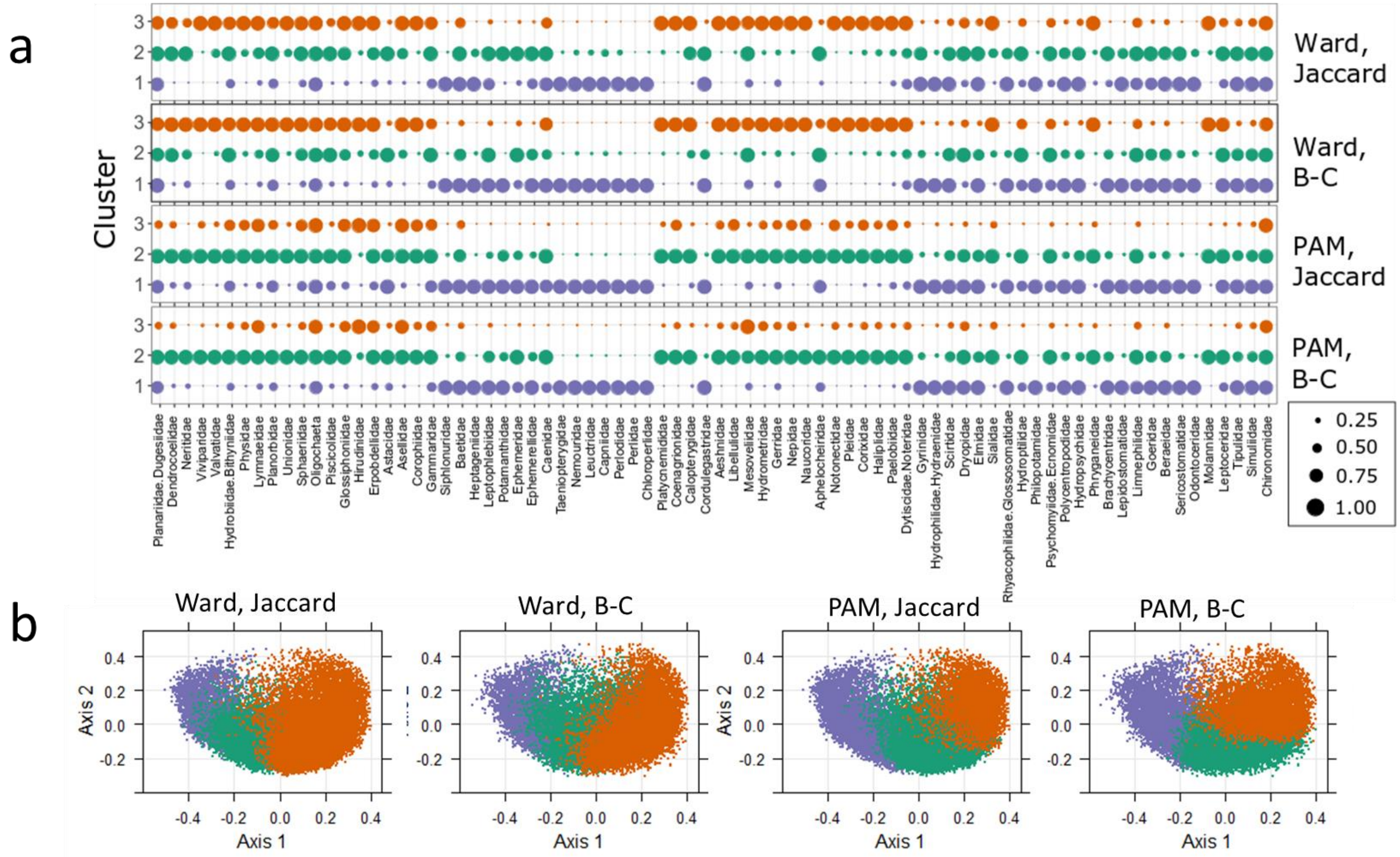

Supplementary Figure 4b. Extended version of Supplementary Figure 4a: the biological classes returned by the four cluster analysis methods (Ward's and partitioning around medoids (PAM) clustering, using Jaccard and Bray-Curtis (B-C) dissimilarities). (**A**) Relative prevalence of the 78 macroinvertebrate taxa across the three classes for each of the four methods (Ward, Jaccard is the same as Supplementary Figure S4a). (**B**) Class membership of the 19,915 samples, plotted on identical principal coordinate analysis axes, calculated using Jaccard dissimilarities (colour scheme same as (**A**)). Source data are provided as a Source Data file.

A

B

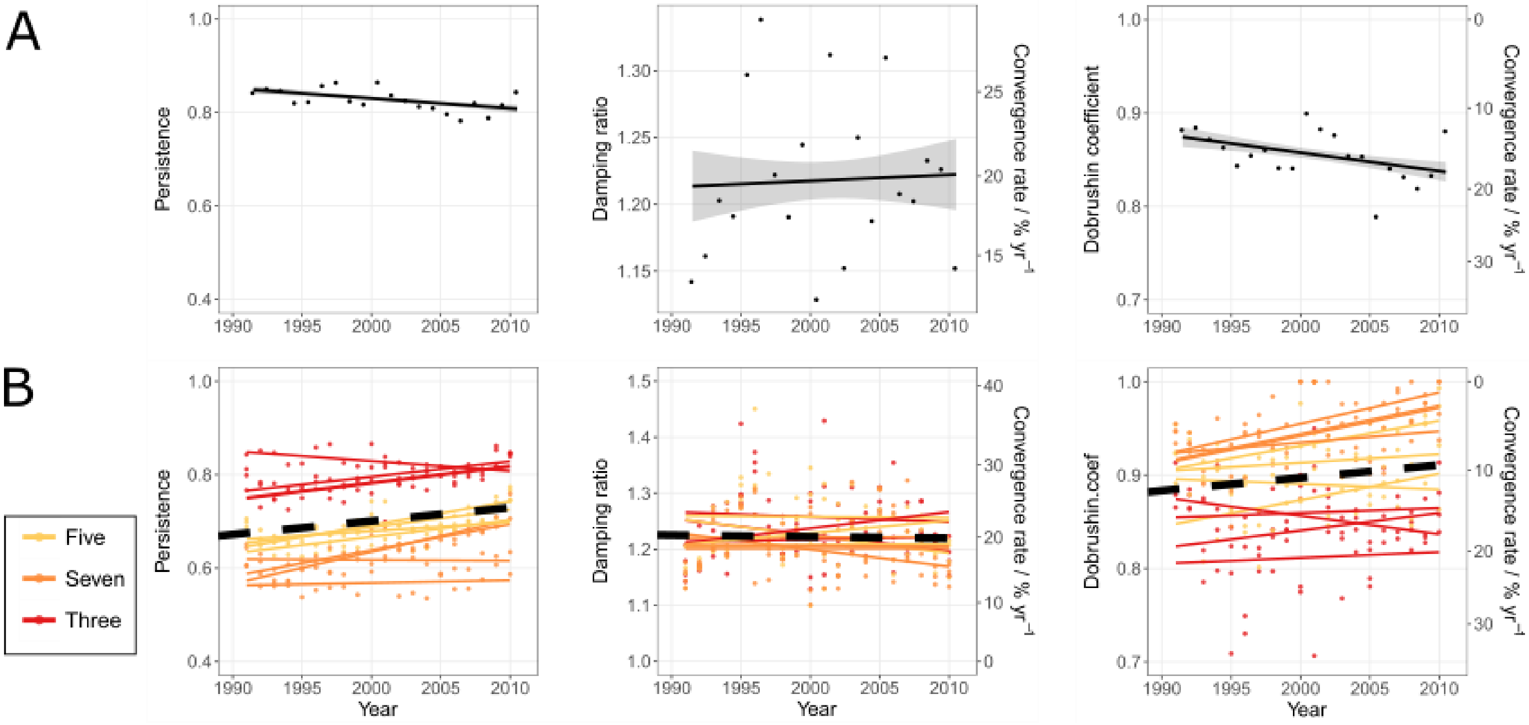

Supplementary Figure 5. Changes through time in three community-level metrics derived from the Markov state transition model for: (**A**) Ward's clustering based on Jaccard distances (three biological classes) and (**B**) all 12 clustering approaches. The three measures are (left to right): the proportion of communities staying in the same class between years (persistence), the damping ratio and Dobrushin's coefficient. Lines in (**A**) are predictions for fitted linear models (± standard error). In **B**, individual regression lines are shown for all 12 clustering approaches, with an overall line (black, dashed) based on a mixed effects models (see Supplementary Table 3 for model outputs) and different colours for the different number of biological classes. Source data are provided as a Source Data file.

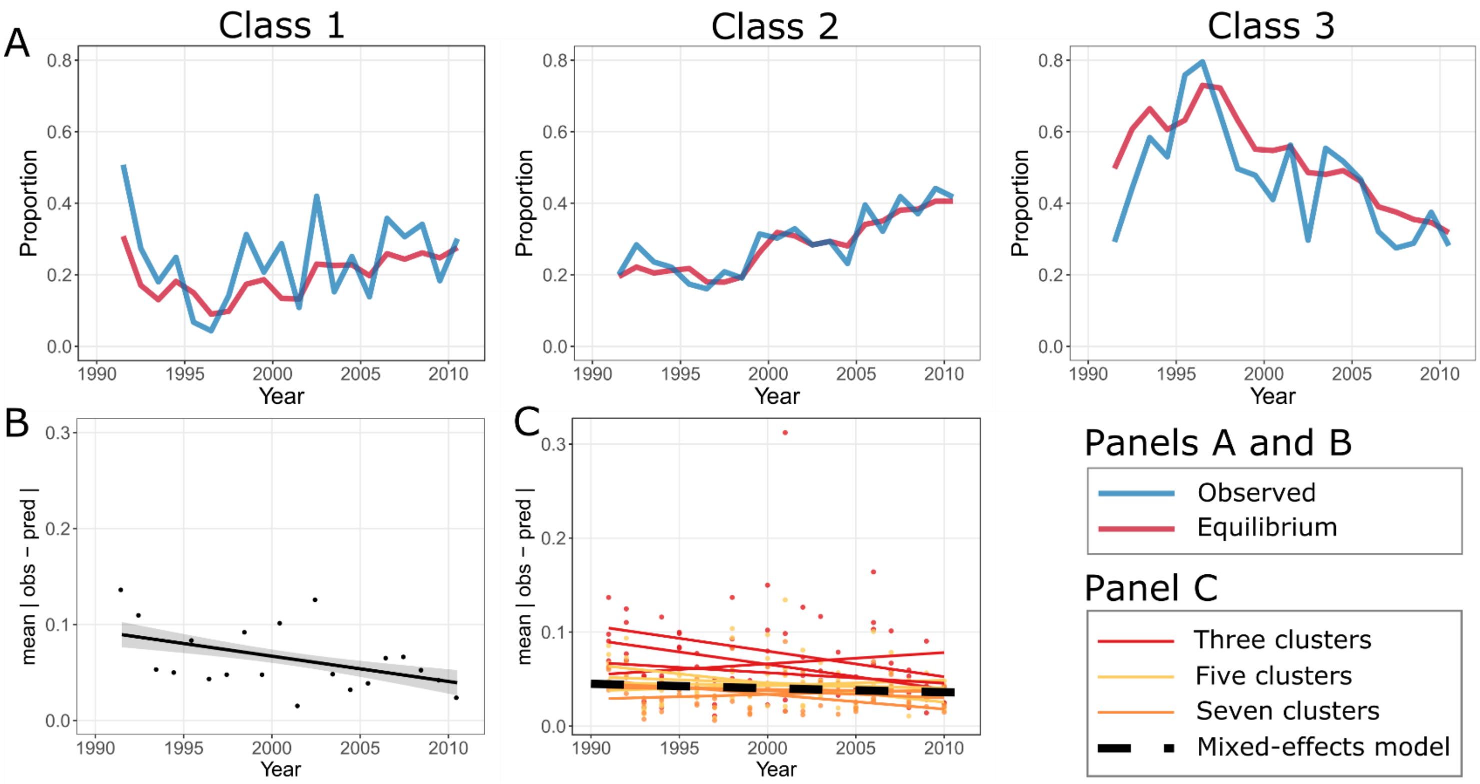

Supplementary Figure 6. Comparisons between the observed and equilibrium prevalence of the biological classes. (**A**) The observed and estimated prevalence at each annual transition for the three classes. Points are plotted at the mid-point of each transition (e.g. 1995-6 is plotted at 1995.5) and use the observed values for the second year of a pair (e.g. 1996 for 1995-6). Note that the proportions are calculated from the locations sampled in each year to allow comparison against the Markov model, rather than adjusting for missing locations (cf. Fig. 1b in main text). (**B**) The mean absolute difference between observed (obs) and equilibrium (pred) prevalence across the three classes. Parts (**A**) and (**B**) present the results for Ward's clustering of Jaccard dissimilarities. (**C**) is equivalent to (**B**), but presents the results for all 12 clustering approaches. Model outputs for (**B**) and (**C**) are in Supplementary Table 3. Source data are provided as a Source Data file.

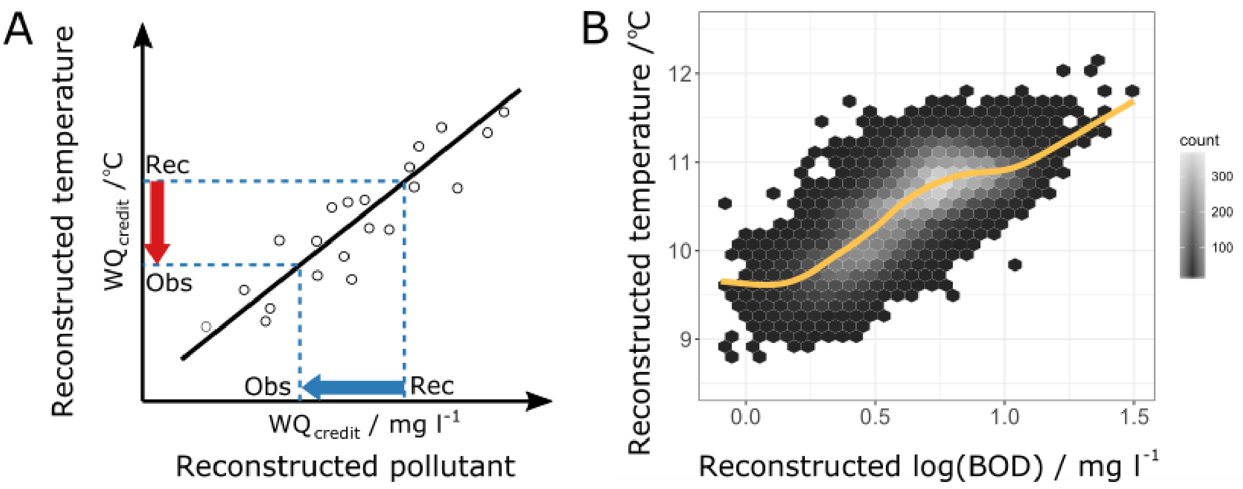

Supplementary Figure 7. Converting changes in water quality to their temperature equivalents. (**A**) Reconstructed water temperatures, inferred from the observed macroinvertebrate communities, are regressed onto the reconstructed pollutant concentrations inferred from the same data. Water quality credit (WQ_{credit}) is defined as the difference between the observed (Obs) and reconstructed (Rec) pollutant concentrations at a point from the time series in Fig. 4b in the main paper. These values can be converted to the equivalent temperatures inferred from the communities, allowing the credit to be expressed in the same units as the climatic debt (i.e. °C). (**B**) The relationship between reconstructed temperatures and biochemical oxygen demand in English and Welsh rivers, based on 27,378 macroinvertebrate samples, summarised using hexagonal binning for clarity. The relationship was modelled using a generalised additive model (orange line; R^2 = 0.59).

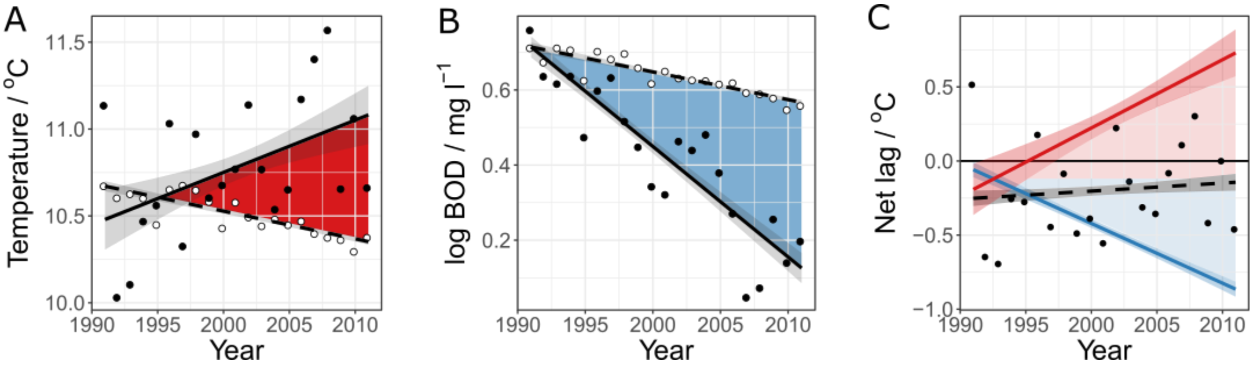

Supplementary Figure 8. Changes in estimated environmental lags across English and Welsh rivers 1991–2011 (± standard error): (**A**) increasing climatic debt and (**B**) water quality credit for BOD, and (**C**) the resulting net environmental lag. The figure is identical to the lower row (panels **D-F**) in Fig. 4 of the main paper, except that the y-intercepts of the GLS fits were not constrained to be identical (**A** and **B**) or set to zero (**C**). Source data are provided as a Source Data file (same data as Fig. 4).

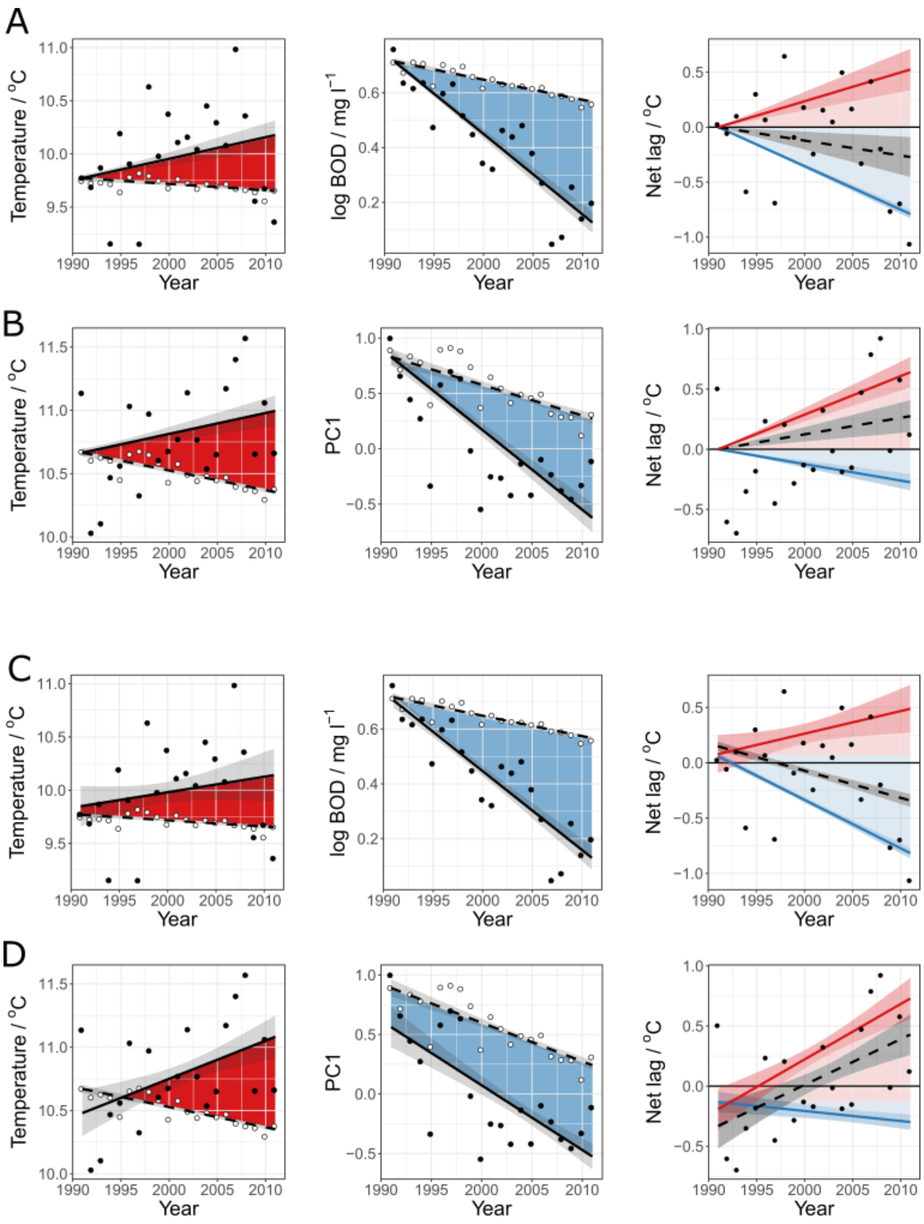

Supplementary Figure 9. Alternative climatic and water quality lags, using both fixed y-intercepts (**A** and **B**) and unconstrained GLS regressions (**C** and **D**). The left hand column portrays the climatic debt, the central column the water quality credit and the right hand column the net environmental lag. (**A**) Air temperature and BOD, (**B**) water temperature and the first principal component (PC1) of nitrate, orthophosphate and discharge; (**C**) and (**D**) are equivalent to (**A**) and (**B**) respectively, but without the constrained y-intercepts. All plotting options (colours and fills) are the same as Fig. 4 (main paper) and Supplementary Figure 8. Source data are provided as a Source Data file.

Supplementary tables

Transition	*n*	Transition	*n*
1991-1992	1400	2001-2002	136
1992-1993	726	2002-2003	817
1993-1994	790	2003-2004	730
1994-1995	985	2004-2005	570
1995-1996	1121	2005-2006	607
1996-1997	677	2006-2007	656
1997-1998	713	2007-2008	554
1998-1999	829	2008-2009	589
1999-2000	1111	2009-2010	606
2000-2001	179	2010-2011	547

Supplementary Table 1. The number of annual transitions used for the Markov chain model (total n = 14,343). The number of transitions was much smaller for 2000-1 and 2001-2, due to limited sampling in 2001.

					Clustering method							
Upland versus lowland rivers					Ward, Jaccard		Ward, Bray-Curtis		PAM, Jaccard		PAM, Bray-Curtis	
Test	Term	Model 1	Model 2	Δdf	ΔG^2	*p*	ΔG^2	*p*	ΔG^2	*p*	ΔG^2	*p*
Time 1	*FT, FST*	*FS, STL*	*FST, STL*	114	344.99	<0.001	382.62	<0.001	383.53	<0.001	484.39	<0.001
Time 2	*FT, FST*	*FSL, STL*	*FST, FSL, STL*	114	345.04	<0.001	402.63	<0.001	367.39	<0.001	471.91	<0.001
Space 1	*FL, FSL*	*FS, STL*	*FSL, STL*	6	256.56	<0.001	312.66	<0.001	199.54	<0.001	286.89	<0.001
Space 2	*FL, FSL*	*FST, STL*	*FST, FSL, STL*	6	256.61	<0.001	332.67	<0.001	183.4	<0.001	274.4	<0.001
T x *S*	*FTL, FTSL*	*FST, FSL, STL*	*FSTL*	114	137.02	0.070	109.94	0.590	131.82	0.122	153.34	0.008
Urban versus rural rivers												
Test	Term	Model 1	Model 2	Δdf	ΔG^2	*p*	ΔG^2	*p*	ΔG^2	*p*	ΔG^2	*p*
Time 1	*FT, FST*	*FS, STL*	*FST, STL*	114	344.99	<0.001	382.62	<0.001	383.53	<0.001	484.39	<0.001
Time 2	*FT, FST*	*FSL, STL*	*FST, FSL, STL*	114	351.23	<0.001	389.01	<0.001	391.01	<0.001	489.77	<0.001
Space 1	*FL, FSL*	*FS, STL*	*FSL, STL*	6	235.45	<0.001	203.37	<0.001	226.72	<0.001	253.51	<0.001
Space 2	*FL, FSL*	*FST, STL*	*FST, FSL, STL*	6	241.69	<0.001	209.76	<0.001	234.20	<0.001	258.89	<0.001
T x *S*	*FTL, FTSL*	*FST, FSL, STL*	*FSTL*	114	130.54	0.138	145.33	0.025	121.60	0.296	156.54	0.005

Supplementary Table 2a. Loglinear analysis results for the four clustering approaches, testing the significance of spatial and temporal variation in transition probabilities by comparing nested models: model notation follows Hill et al.[2]. The first half of the table compares upland and lowland rivers, and the second half compares rural and urban rivers. There are two pairs of models that allow a test of the space and time terms respectively, which are labelled as Space 1 and 2, and Time 1 and 2. *T* x *S* denotes the interaction between space and time and ΔG^2 is the change in the likelihood (goodness of fit).

			Clustering approach											
Upland versus lowland rivers			Ward, Jaccard			Ward, Bray-Curtis			PAM, Jaccard			PAM, Bray-Curtis		
Model		df	G^2	AIC	ΔAIC	G^2	AIC	ΔAIC	G^2	AIC	ΔAIC	G^2	AIC	ΔAIC
Null	*FS, STL*	234	738.62	270.62	361.6	825.23	357.23	475.29	698.75	230.75	326.93	912.13	444.13	518.79
T-varying	*FST, STL*	120	393.63	153.63	244.61	442.61	202.61	320.67	315.22	75.22	171.4	427.74	187.74	262.4
S-varying	*FSL, STL*	228	482.06	26.06	117.04	512.57	56.57	174.63	499.21	43.21	139.39	625.25	169.25	243.91
T + *S*	*FST, FSL, STL*	114	137.02	-90.98	0	109.94	-118.06	0	131.82	-96.18	0	153.34	-74.66	0
T x *S*	*FSTL*	0	0	0	90.98	0	0	118.06	0	0	96.18	0	0	74.66
Urban versus rural rivers														
Model		df	G^2	AIC	ΔAIC	G^2	AIC	ΔAIC	G^2	AIC	ΔAIC	G^2	AIC	ΔAIC
Null	*FS, STL*	234	717.22	249.22	346.68	737.71	269.71	352.38	739.33	271.33	377.73	899.82	431.82	503.28
T-varying	*FST, STL*	120	372.23	132.23	229.69	355.09	115.09	197.76	355.8	115.8	222.2	415.43	175.43	246.89
S-varying	*FSL, STL*	228	481.76	25.76	123.22	534.34	78.34	161.01	512.61	56.61	163.01	646.31	190.31	261.77
T + *S*	*FST, FSL, STL*	114	130.54	-97.46	0	145.33	-82.67	0	121.6	-106.4	0	156.54	-71.46	0
T x *S*	*FSTL*	0	0	0	97.46	0	0	82.67	0	0	106.4	0	0	71.46

Supplementary Table 2b. Comparison of the five models fitted to the transition probabilities for the four clustering approaches, both for upland and lowland, and rural and urban, rivers. Notation follows Hill et al.[2]. The five models are: i) Null = constant transition probabilities over space and time; ii) *T*-varying = transition probabilities identical in the two river types but allowed to vary through time; iii) *S*-varying = constant through time, but varying between the two river types; iv) *T* + *S* = transition probabilities can vary in time and space additively; and v) the most complex model (*T* x *S*), with an interaction between spatial and temporal variability.

Variable	Intercept			Slope		
	Estimate (standard error)	*t*-value	*p*-value	Estimate (standard error)	*t*-value	*p*-value
A. Three-class, hierarchical clustering						
Equilibrium	0.093 (0.015)	6.342	<0.0001	-0.003 (0.001)	2.225	0.0391
Persistence	0.852 (0.010)	86.861	<0.0001	-0.002 (0.001)	2.708	0.0144
Damping coefficient	1.213 (0.030)	40.854	<0.0001	0.000 (0.002)	0.192	0.8500
Dobrushin's coefficient	0.877 (0.012)	74.780	<0.0001	-0.002 (0.001)	2.080	0.0520
B. Mixed effects models for all 12 clustering approaches						
Equilibrium	-3.101 (0.123)	25.212	<0.0001	-0.011 (0.008)	-1.469	0.1431
Persistence	0.672 (0.025)	26.542	<0.0001	0.003 (0.001)	4.109	0.0001
Damping coefficient	1.225 (0.010)	122.834	<0.0001	-0.000 (0.001)	-0.355	0.7229
Dobrushin's coefficient	0.883 (0.015)	59.846	<0.0001	0.001 (0.001)	2.620	0.0094

Supplementary Table 3. Regression results for changes through time in four Markov model derived metrics: i) the mean absolute difference between the observed prevalence of each class and the estimated prevalence at equilibrium (= Equilibrium), ii) the proportion of communities remaining in the same class between years (= Persistence), iii) the damping coefficient and iv) Dobrushin's coefficient. Results are presented for: (**A**) the main three-cluster analysis (Ward's method, Jaccard dissimilarities), with models fitted using GLS; (**B**) all 12 clustering approaches, fitted using a linear mixed-effects model. Results are plotted in Supplementary Figure 5. Degrees of freedom for *t*-values are 18 (Part A) and 227 (Part B).

State	Fate	Coefficient estimate (standard error)	*t*-value	*p*-value
1	1	0.003 (0.003)	1.011	0.3252
1	2	-0.001 (0.002)	0.408	0.6883
1	3	-0.001 (0.003)	0.412	0.6855
2	1	-0.004 (0.002)	2.126	0.0476
2	2	0.008 (0.002)	4.180	<0.0001
2	3	-0.002 (0.005)	0.402	0.6924
3	1	-0.000 (0.000)	0.014	0.9891
3	2	0.005 (0.001)	3.988	<0.0001
3	3	-0.005 (0.001)	3.549	0.0023

Supplementary Table 4. Slope coefficients for the generalised least squares models of transition probabilities regressed on time (accompanies Fig. 2 in the main text). Nine outcomes are possible, either staying in the same class (three types) or moving among classes (six types). Degrees of freedom for *t*-values is 18. Source data are provided as a Source Data file (same data as for Fig. 2 in the main paper).

Response variable	Intercept			Year			Interaction		
	Estimate (standard error)	*t*-value	*p*-value	Estimate (SE)	*t*-value	*p*-value	Estimate (standard error)	*t*-value	*p*-value
Observed v. reconstructed conditions (df = 39)									
T_{water}	10.66 (0.03)	407.41	<0.0001	0.02 (0.01)	2.35	0.0239	-0.03 (0.01)	4.68	<0.0001
BOD	0.71 (0.01)	79.89	<0.0001	-0.03 (0.00)	16.33	<0.0001	0.02 (0.00)	12.76	<0.0001
T_{air}	9.77 (0.02)	468.20	<0.0001	0.02 (0.01)	2.34	0.0247	-0.03 (0.01)	3.05	0.0041
Nutrients	0.83 (0.08)	10.82	<0.0001	-0.07 (0.01)	7.43	<0.0001	0.04 (0.01)	5.01	<0.0001
ii) Environmental lags (df = 20)									
T_{water}	–	–	–	0.03 (0.01)	4.74	0.0001	–	–	–
T_{air}	–	–	–	0.03 (0.01)	3.08	0.0060	–	–	–
BOD (°C; water)	–	–	–	-0.04 (0.00)	12.50	<0.0001	–	–	–
BOD (°C; air)	–	–	–	-0.04 (0.00)	11.18	<0.0001	–	–	–
Nutrients (°C; water)	–	–	–	-0.01 (0.01)	2.06	0.0530	–	–	–
iii) Net environmental lags (df = 20)									
T_{water} v. BOD	–	–	–	-0.01 (0.01)	1.97	0.062	–	–	–
T_{water} v. nutrients	–	–	–	0.01 (0.01)	1.80	0.087	–	–	–
T_{air} v. BOD	–	–	–	-0.01 (0.01)	1.69	0.107	–	–	–

Supplementary Table 5a. Fitted GLS models for: i) comparing observed and reconstructed environmental conditions, ii) environmental lags for individual variables, and iii) net lags for paired environment variables (regression fits are shown in Fig. 4 and Supplementary Figure 9). All models have fixed y-intercepts, either a common value for the observed and reconstructed conditions (i) or zero (ii and iii). The four environmental variables are water temperature (T_{water}), BOD, air temperature (T_{air}), and the first principal component of discharge, nitrate and orthophosphate (= ‘Nutrients’). For modelling the environmental lags, BOD and nutrients were first converted to equivalent water or air temperature changes (denoted by ‘°C; water’ or ‘°C; air’ respectively. Source data are provided as a Source Data file.

Response variable	Intercept			Time series: Reconstructed			Year			Interaction		
	Estimate	*t*-value	*p*-value	Estimate	*t*-value	*p*-value	Estimate	*t*-value	*p*-value	Estimate	*t*-value	*p*-value
i) Free intercept models (df = 38)												
T_{water}	10.48 (0.15)	69.75	<0.0001	0.19 (0.15)	1.26	0.2166	0.03 (0.01)	2.343	0.0245	-0.05 (0.01)	3.53	0.0011
BOD	0.71 (0.04)	18.19	<0.0001	0.01 (0.04)	0.25	0.8033	-0.03 (0.00)	8.667	<0.0001	0.02 (0.00)	6.26	<0.0001
T_{air}	9.85 (0.20)	49.92	<0.0001	-0.08 (0.20)	0.40	0.6926	0.02 (0.02)	0.865	0.3926	-0.02 (0.02)	1.21	0.2357
Nutrients	0.56 (0.14)	4.01	0.0003	0.33 (0.15)	2.15	0.0378	-0.05 (0.01)	4.534	0.0001	0.02 (0.01)	1.68	0.1021
ii) *Environmental lags (df = 19)*												
T_{water}	-0.19 (0.15)	1.28	0.218	–	–	–	0.05 (0.01)	3.58	0.0020	–	–	–
BOD (°C; water)	-0.06 (0.08)	0.72	0.479	–	–	–	-0.04 (0.01)	5.85	<0.0001	–	–	–
T_{air}	0.08 (0.20)	0.40	0.692	–	–	–	0.02 (0.02)	1.22	0.2390	–	–	–
BOD (°C; air)	0.06 (0.08)	0.76	0.460	–	–	–	-0.04 (0.01)	6.51	<0.0001	–	–	–
Nutrients (°C; water)	-0.13 (0.05)	1.28	0.218	–	–	–	0.05 (0.01)	3.58	0.0020	–	–	–
iii) Net environmental lags (df = 19)												
T_{water} v. BOD	-0.25 (0.14)	1.78	0.0908	–	–	–	0.01 (0.01)	0.45	0.6592	–	–	–
T_{water} v. nutrients	-0.33 (0.16)	2.07	0.0526	–	–	–	0.04 (0.01)	2.78	0.0121	–	–	–
T_{air} v. BOD	0.15 (0.18)	0.82	0.4242	–	–	–	-0.02 (0.02)	1.57	0.1338	–	–	–

Supplementary Table 5b. Equivalent models to those in Supplementary Table S5a, comparing observed and reconstructed environmental conditions and estimating environmental lags, but with no constraints placed on the y-intercepts of the GLS models. Models fits are displayed in Supplementary Figures 8 and 9. Source data are provided as a Source Data file.

Supplementary references

1. Caswell, H. *Matrix Population Models* (2nd edition). Sinauer Associates, Sunderland, MA (2001).
2. Hill, M. F., Witman, J. D. & Caswell, H. Spatio-temporal variation in Markov chain models of subtidal community succession. *Ecol. Lett.* **5,** 665–675 (2002).
3. Vaughan, I. P. & Ormerod, S. J. Large-scale, long-term trends in British river macroinvertebrates. *Glob. Change Biol.* **18,** 2184–2194 (2012).
4. Fuller, R. M. *et al*. The UK land cover map 2000: planning, construction and calibration of a remotely sensed, user-oriented map of broad habitats. *Int. J. Appl. Earth Obs.* **7,** 202–216 (2005).
5. Vaughan, I. P. & Ormerod, S. J. Linking interdecadal changes in British river ecosystems to water quality and climate dynamics. *Glob. Change Biol*. **20,** 2725–2740 (2014).
6. Extence, C. A., Balbi, D. M. & Chadd, R. P. River flow indexing using British benthic macroinvertebrates: a framework for setting hydroecological objectives. *Regul. Riv.* **15,** 543–574 (1999).
7. Armitage, P. D., Moss, D., Wright, J. F. & Furse, M. T. The performance of a new biological water quality score system based on macroinvertebrates over a wide range of unpolluted running-water sites. *Water Res.* **17,** 333–347 (1983).
