# Supplementary material for: Water quality improvements offset the climatic debt for stream macroinvertebrates over twenty years
Source: Nat Commun. 2019 Apr 26;10:1956. doi: 10.1038/s41467-019-09736-3 (PMC6486586; doi:10.1038/s41467-019-09736-3)
Supplement: Supplementary file 3 — Reporting Summary [file 41467_2019_9736_MOESM3_ESM.pdf]

nature research

Corresponding author(s): Ian Vaughan

Reporting Summary

Nature Research wishes to improve the reproducibility of the work that we publish. This form provides structure for consistency and transparency in reporting. For further information on Nature Research policies, see Authors & Referees and the Editorial Policy Checklist.

Statistical parameters

When statistical analyses are reported, confirm that the following items are present in the relevant location (e.g. figure legend, table legend, main text, or Methods section).

n/a	Confirmed	
☐	☒	The exact sample size (n) for each experimental group/condition, given as a discrete number and unit of measurement
☐	☒	An indication of whether measurements were taken from distinct samples or whether the same sample was measured repeatedly
☐	☒	The statistical test(s) used AND whether they are one- or two-sided<br>*Only common tests should be described solely by name; describe more complex techniques in the Methods section.*
☐	☒	A description of all covariates tested
☐	☒	A description of any assumptions or corrections, such as tests of normality and adjustment for multiple comparisons
☐	☒	A full description of the statistics including central tendency (e.g. means) or other basic estimates (e.g. regression coefficient) AND variation (e.g. standard deviation) or associated estimates of uncertainty (e.g. confidence intervals)
☐	☒	For null hypothesis testing, the test statistic (e.g. F, t, r) with confidence intervals, effect sizes, degrees of freedom and P value noted<br>*Give P values as exact values whenever suitable.*
☒	☐	For Bayesian analysis, information on the choice of priors and Markov chain Monte Carlo settings
☒	☐	For hierarchical and complex designs, identification of the appropriate level for tests and full reporting of outcomes
☐	☒	Estimates of effect sizes (e.g. Cohen's d, Pearson's r), indicating how they were calculated
☐	☒	Clearly defined error bars<br>*State explicitly what error bars represent (e.g. SD, SE, CI)*

Our web collection on statistics for biologists may be useful.

Software and code

Policy information about availability of computer code

Data collection	ArcGIS v10 used to estimate urban land cover within each river catchment. All other data were from existing databases.
Data analysis	All analyses were run in R v3.4. All R packages used in the analyses are detailed - with references - in the Methods. Upon acceptance, the full code (R script files) for all of the analyses will be archived alongside the data on GitHub.

For manuscripts utilizing custom algorithms or software that are central to the research but not yet described in published literature, software must be made available to editors/reviewers upon request. We strongly encourage code deposition in a community repository (e.g. GitHub). See the Nature Research guidelines for submitting code & software for further information.

Data

Policy information about availability of data

All manuscripts must include a data availability statement. This statement should provide the following information, where applicable:

- Accession codes, unique identifiers, or web links for publicly available datasets
- A list of figures that have associated raw data
- A description of any restrictions on data availability

Upon acceptance, the complete data set will be archived on GitHub alongside the R code to reproduce the analyses.

Field-specific reporting

Please select the best fit for your research. If you are not sure, read the appropriate sections before making your selection.

☐ Life sciences ☐ Behavioural & social sciences ☒ Ecological, evolutionary & environmental sciences

For a reference copy of the document with all sections, see nature.com/authors/policies/ReportingSummary-flat.pdf

Ecological, evolutionary & environmental sciences study design

All studies must disclose on these points even when the disclosure is negative.

Study description

The study comprises two parts, using macroinvertebrate community data collected from rivers across England and Wales (n = 3067 locations):

1. Using a Markov chain model to reveal how dynamic properties of river macroinvertebrate communities changed through time and estimate the proximity of the observed communities to equilibrium with their environment (n = 19,915 samples, representing 14,343 annual transitions). Changes in properties such as asymptotic stability (damping ratio) and proximity to equilibrium were regressed onto year to assess evidence for change through time.

2. Using transfer functions to estimate the extent of the climatic debt, water quality 'credit' and net environmental lag from the same 3067 locations (n = 27,378). As in (1), debts, credits and net lags were regressed onto year to assess changes through time.

Research sample

The data were supplied by the Environment Agency, the statutory agency monitoring rivers in England and Wales over the 1991-2011 study period. Locations across England and Wales (n = 3067) were sampled repeatedly across the study period. A quality assurance programme was in place during the study period, and indicated a very high level of consistency through time (details in the Methods and references therein).

Sampling strategy

We used the largest possible number of sampling locations from the Environment Agency database, subject to the following screening procedure:

1. Locations were removed if they were immediately downstream of wastewater or industrial outfalls, and/or had not been sampled in at least one pair of consecutive years during the study period (necessary for calculating annual transition probabilities).

2. To provide more even geographic coverage across England and Wales, we selected one location from each river catchment (see Methods for full details).

All data screening was carried out before the data analysis commenced.

Data collection

Samples were collected by Environment Agency staff, using a standardised 3-minute kick sampling protocol, with sorting and identification carried out in laboratories with a QA procedure in place to ensure consistency over time.

Timing and spatial scale

Samples were collected 1991-2011 inclusive in Spring (March-May inclusive). Sampling frequency varied among the 3067 locations, ranging from 2-21 years sampled (mean = 8.9 samples per location).

Data exclusions

Some sampling locations were omitted prior to data analysis commencing (see details in 'Sampling strategy' above)

Reproducibility

Key analyses were repeated to assess the sensitivity of the results to the choices made. These are described in the main text and Methods. Briefly, there were two main areas:

1. Cluster analysis was used to classify the biological data, which in turn formed the basis for the Markov chain analyses. The clustering, and subsequent analyses (Markov chain and regressions), were re-run using four different clustering methods and three different classifications (3, 5 or 7 clusters), resulting in 12 permutations in total. These are presented in the results/supplementary material and confirmed that the results were robust to the choice of approach.

2. The core climate debt-water quality credit analysis presented in the paper was repeated: i) using air, rather than water, temperatures, and ii) nutrient concentrations (cf. Biochemical Oxygen Demand). Full results are presented in the main text and supplementary material, and indicate qualitatively similar outcomes, albeit with different magnitudes of the estimated credits, debts and lags.

Randomization

There was no allocation of subjects to groups.

Blinding

Blinding was not relevant to this study because no experimental intervention was used and the data had already been collected.

Did the study involve field work? ☐ Yes ☒ No

Reporting for specific materials, systems and methods

Materials & experimental systems

n/a	Involved in the study
☒	☐ Unique biological materials
☒	☐ Antibodies
☒	☐ Eukaryotic cell lines
☒	☐ Palaeontology
☐	☒ Animals and other organisms
☒	☐ Human research participants

Methods

n/a	Involved in the study
☒	☐ ChIP-seq
☒	☐ Flow cytometry
☒	☐ MRI-based neuroimaging

Animals and other organisms

Policy information about studies involving animals; ARRIVE guidelines recommended for reporting animal research

Laboratory animals	No laboratory animals.
Wild animals	Macroinvertebrates were sampled using a standard 3-minute kick sampling protocol and preserved in 70% ethanol.
Field-collected samples	Macroinvertebrates were sampled using a standard 3-minute kick sampling protocol and preserved in 70% ethanol.
